# Wellbeing in Secondary Education (WISE) Study to Improve the Mental Health and Wellbeing of Teachers: A Complex System Approach to Understanding Intervention Acceptability

**DOI:** 10.1007/s11121-022-01351-x

**Published:** 2022-03-19

**Authors:** Rhiannon Evans, Sarah Bell, Rowan Brockman, Rona Campbell, Lauren Copeland, Harriet Fisher, Tamsin Ford, Sarah Harding, Jillian Powell, Nicholas Turner, Judi Kidger

**Affiliations:** 1 DECIPHer, School of Social Sciences, Cardiff University, 1-3 Museum Place, Cardiff, CF10 3BD Wales; 2 Bristol Medical School, University of Bristol, Bristol, England; 3 Department of Population Health Sciences, University of Bristol, Bristol, England; 4 Department of Psychiatry, University of Cambridge, Cambridge, England; 5 School for Policy Studies, University of Bristol, Bristol, England

**Keywords:** Adolescents, Teacher, School, Process evaluation, Mental health

## Abstract

Teaching staff report poorer mental health and wellbeing than the general working population. Intervention to address this issue is imperative, as poor wellbeing is associated with burnout, presenteeism, and adverse student mental health outcomes. The Wellbeing in Secondary Education (WISE) intervention is a secondary school-based programme aimed at improving the mental health and wellbeing of teachers and students. There are three components: awareness-raising for staff; a peer support service delivered by staff trained in Mental Health First Aid (MHFA); and Schools and Colleges Mental Health First Aid (MHFA) training for teachers. A cluster randomised controlled trial with integrated process and economic evaluation was conducted with 25 secondary schools in the UK (2016–2018). The intervention was largely ineffective in improving teacher mental health and wellbeing. This paper reports process evaluation data on acceptability to help understand this outcome. It adopts a complex systems perspective, exploring how acceptability is a dynamic and contextually contingent concept. Data sources were as follows: interviews with funders (*n* = 3); interviews with MHFA trainers (*n* = 6); focus groups with peer supporters (*n* = 8); interviews with headteachers (*n* = 12); and focus groups with teachers trained in Schools and Colleges MHFA (*n* = 7). Results indicated that WISE intervention components were largely acceptable. Initially, the school system was responsive, as it had reached a ‘tipping point’ and was prepared to address teacher mental health. However, as the intervention interacted with the complexities of the school context, acceptability became more ambiguous. The intervention was seen to be largely inadequate in addressing the structural determinants of teacher mental health and wellbeing (e.g. complex student and staff needs, workload, and system culture). Future teacher mental health interventions need to focus on coupling skills training and support with whole school elements that tackle the systemic drivers of the problem.

## Introduction

The mental health and wellbeing of teachers is an important public health concern. The UK data show that they have a higher prevalence of reported cases of work-related stress, anxiety, and depression compared to other professions (Health & Safety Executive, 2019). This may have detrimental impacts on presenteeism (attending work despite poor health), sickness absence, and withdrawal from the profession (Henderson et al., 2011; Kidger et al., 2016a). Causes include unmanageable workloads, lack of autonomy, difficult relationships with colleagues, and a stringent culture of regulation and performance management (Barmby, 2006; Skaalvik & Skaalvik, 2017). Attending to teachers’ mental health is important for student mental health and wellbeing (Harding et al., 2019). Evidence reports that teacher wellbeing is associated with better student wellbeing and with fewer student psychological difficulties. These associations are explained through the mediating factors of teacher presenteeism and teacher-student relationships (Harding et al., 2019; Jennings et al., 2011).

Studies have examined the impact of workplace interventions on mental health. Effectiveness is reported for cognitive behavioural therapy, self-help, exercise, and mindfulness (Bartlett et al., 2019; Nigatu et al., 2019). There is an increasing number of interventions for teachers, although these tend to be individually focused and rarely address the structural drivers of poor mental health (Iancu et al., 2018; Oliveira et al., 2021; von der Embse et al., 2019). Fostering workplace peer support is a potential approach, as schools can be seen as having unsupportive environments (Kidger et al., 2010). In other professions, peer support has been shown to create a supportive culture, while avoiding the perceived stigma attached to more formal help sources (Linnan et al., 2013).

Mental Health First Aid (MHFA) is an internationally recognised intervention that can offer peer support (Kitchener & Jorm, 2004). It aims to improve knowledge of the signs and symptoms of mental disorders and enhance confidence to help those in crisis. The training has been shown to impact confidence, knowledge, and skills, although effects on the mental health of those receiving support has not been fully established (Jorm et al., 2010; Kelly et al., 2011). The original ‘standard’ MHFA package is for adults supporting adults, but a one day version specifically for school staff supporting students was subsequently designed (Jorm et al., 2010). A version called MHFA for Schools and Colleges has been developed for use in educational settings in England, primarily in secondary schools.

The present paper reports findings from a process evaluation of the Wellbeing in Secondary Education (WISE) intervention, which aims to improve the mental health and wellbeing of teachers and students. The overarching study was a cluster randomised controlled trial with an integrated process and economic evaluation, conducted across twenty-five secondary schools in England and Wales (2016–2018) (Kidger et al., 2016b). A previous pilot and feasibility trial indicated that the intervention was relevant and acceptable to school staff (Kidger et al., 2016b). However, the effectiveness trial found limited impact on teacher mental health and wellbeing (Kidger et al., 2021). The reported analysis examines intervention acceptability, addressing the research question: Is the WISE intervention acceptable to funding organisations, intervention trainers, head-teachers, teachers, and students? These data help to explain the lack of intervention effectiveness.

While the construct of acceptability is commonly explored in process evaluations, it has received limited conceptual development. Definitions and frameworks have focused on the appraisal of intervention components assessing discrete and often quantifiable constructs, such as: affective attitude; burden; ethicality; intervention coherency; opportunity cost; perceived effectiveness; and self-efficacy for delivery (Sekhon et al., 2017). In contrast, Medical Research Council funded guidance on process evaluations has considered acceptability more of a dynamic experience, focusing on the evolving interaction of participant and intervention within a complex set of contextual conditions (Moore et al., 2015). This framing reflects ongoing advances in complex system perspectives (Hawe et al., 2009; Moore et al., 2018), which state that the effectiveness of an intervention is contingent on the antecedent and emergent properties of the system in which it is implemented. While the WISE study used constructs from extant frameworks to assess perceptions of intervention components (Sekhon et al., 2017), it foregrounded the conceptual approach to acceptability outlined by the process evaluation guidance (Moore et al., 2015). As such, it sought to qualitatively understand participants’ changing experience of the intervention, and how this was shaped by wider system dynamics.

## Wellbeing in Secondary Education Intervention (WISE)

The Wellbeing in Secondary Education (WISE) intervention comprised three components. The intervention as a whole was newly developed, but integrated existing MHFA training packages in order to develop staff skills. More detail is reported in the study protocols and related publications (Fisher et al., 2020; Kidger et al., 2016b), while further information on the MHFA training package can be found at mhfaengland.org. The intervention is underpinned by the theory of social support (Thoits, 2011). Support that offers problem-focused coping strategies and emotion-focused supportive strategies can have a positive impact on physical and mental health. The MHFA training package supports participants to recognise the signs of poor mental health, and develop the skills and confidence to empower someone to seek help.

### School Staff Awareness-Raising Session

All school staff were offered a 1-hour awareness-raising session. It highlighted the importance of mental health in schools, provided advice on how to support mental health, and introduced the peer support service. The session was designed in collaboration between the study team and MHFA trainers.

### Staff Peer Support Service

Teaching and support staff were nominated by colleagues in the outcome evaluation baseline survey, where respondents listed the three colleagues they were most likely to seek support from. In total, 8% of staff, with a maximum of 16, attended the 2-day standard MHFA training. These included the most nominated staff, while ensuring a balance in socio-demographic profile and role. For example, if a high percentage of individuals who identified as female were nominated, those receiving a slightly lower number of nominations were replaced with the most frequently nominated individuals identifying as male. Trained staff established a confidential peer support service for colleagues, offering informal support and signposting to other services.

### Teacher Training in MHFA for Schools and Colleges

Teachers (an additional 8% of teaching staff with a maximum of 16), selected by the Senior Leadership Team, attended the one-day MHFA for Schools and Colleges training.

Training for the three components was delivered by MHFA instructors in England (*n* = 3) and Healthy School Coordinators (HSCs) in Wales (*n* = 7). HSCs deliver the World Health Organisation’s Health Promoting School Framework, providing wellbeing activities within the school context. HSCs were trained by certified MHFA instructors, and were identified for delivery by the intervention funder in order to develop a sustainable delivery model in Wales.

## Methodology

### Study Design

A mixed-method process evaluation was undertaken, with further detail reported in the protocol (Evans et al., 2018). Acceptability was explored as part of the process evaluation.

### Sample and Data Sources

Twenty-five schools participated in the study with twelve schools receiving the intervention and 13 being control schools. All schools provided process evaluation data, with more in-depth data provided by eight case study schools (four intervention, four control). They were purposively sampled to achieve variation in geographical area, free school meal eligibility, and rating by school inspectorate. Participant sample sizes in each school, and across each data source, were guided by the principle of saturation, with the aim of achieving conceptual depth and representation across a number of schools and stakeholders. Data on intervention acceptability were collected from the 12 intervention schools, which included the four intervention case study schools. Only data from these schools are included in this paper, along with data from funders. 

Topic guides explored participants’ perceptions and experiences, with each guide including specific questions about the potential positive and negative aspects of the intervention. Additional questions were asked about the school context. Some qualitative data on the acceptability of the training, generated through peer supporter logs and post-training surveys, are considered in a related publication on intervention implementation (Fisher et al., 2020).

The presented data were collected between June 2016 and July 2018. Collection was conducted in two phases, exploring changes in perceptions as implementation progressed. Phase 1 was undertaken June 2016–July 2017, after the delivery of intervention training to schools. Training was delivered between September–December 2016, with some sessions delivered in January 2017 due to scheduling challenges. Where possible, training was delivered as early as possible during the autumn term. Phase 2 was conducted between September 2017–July 2018, after the intervention had been delivered for approximately one year.

#### Interviews with Intervention Funding Organisation Representatives

Semi-structured telephone interviews were conducted with representatives from the three organisations that contributed to intervention costs (*n* = 3). These were Bristol City Council, Public Health England and Public Health Wales. Interviews were undertaken during Phase 1.

#### Interviews with MHFA Instructors

Semi-structured interviews were conducted with three MHFA instructors in England and three of the seven HSCs in Wales. HSCs were purposively sampled to ensure the training courses at each of the six Welsh intervention schools were represented. Interviews were undertaken during Phase 1.

#### Interviews with School Headteachers

Semi-structured interviews were conducted with headteachers across the 12 intervention schools. Interviews were conducted at the school site and undertaken during Phase 1.

#### Focus Groups with Peer Supporters (Trained in MHFA)

Focus groups were conducted with peer supporters at each of the four intervention case study schools. All peer supporters were invited to attend, with four to eight participants taking part in each school based on availability. One focus group per school was conducted in person during Phase 1, shortly after training, and again during Phase 2, following approximately a year of delivery.

#### Focus Groups and Interviews with Teachers (Trained in MHFA for Schools and Colleges)

Focus groups were undertaken with teachers who had attended the MHFA for Schools and Colleges training at each of the four intervention case study schools. All attending teachers were invited to participate, with four to eight individuals attending. One focus group was conducted in person at each school site during Phase 1 and one during Phase 2. During Phase 2, individual interviews were conducted in one school due to logistical challenges.

### Analysis

Focus groups and interviews were audio-recorded, transcribed verbatim by a professional transcription service, and anonymised. Thematic analysis was conducted by eight researchers (Braun & Clarke, 2006) on completion of Phase 1 (2016–2017) and Phase 2 (2017–2018). A subset of data was indexed by two researchers to generate a priori and in vivo codes, which were agreed by the team. The codes from Phase 1 were further developed and expanded upon during the second phase. Codes were presented in a codebook, which allowed the team to work with a shared understanding of their meanings. Remaining transcripts were coded by one researcher, with a second researcher coding 10% to check interpretation and reliability. The codebook was flexible and codes were added and adapted until all data had been analysed. Following coding, the research team generated themes at each timepoint by comparing and contrasting codes within and across data sources. Themes were identified according to their ‘keyness’, which was understood as a relevance to the research questions and significance to participants, as signalled by the extent and depth of discussion in relation to them (Braun & Clarke, 2006). The result sections indicate if themes were predominant in specific data sources or common across them. On completion of the phases of data analysis, themes were assessed to trace any evolution during the study. NVivo 11 supported data management and analysis. The data presented are linked to the specific theme of acceptability and the quotes included are typical responses from participants.

## Results

Results are presented in two sections. First, they explore stakeholders’ perceptions of the WISE intervention components. These were largely positive views in relation to intervention coherency and comprehensibility, perceived self-efficacy, and effectiveness. Second, they consider the interaction of intervention components and the system, and how this led to a more complex and ambiguous sense of acceptability. Despite school systems wanting to address staff wellbeing, the intervention was increasingly considered as minimally disruptive of the structural determinants of poor mental health. There determinants were as follows: complex student and staff needs; an organisational culture of mistrust; and a challenging workload and accountability structure. The themes and subthemes related to these two sections are presented in Table 1.

### Acceptability of WISE Intervention Components

Stakeholders from schools who received the WISE intervention generally found components to be acceptable from the outset, suggesting an overarching positive affective attitude:

“Yes, I mean staff were really excited, they were really bubbly, they were really enthusiastic about it. And they’ve had a couple of meetings as a group of staff to talk about, right, we’ve done the training, it was brilliant, really useful, how are we going to impact on staff, as well as on students? (Headteacher, School 2L England)”

There were three key dimensions of acceptability discussed, when mapping the data against existing conceptual frameworks (Sekhon et al., 2017). First was intervention coherency and comprehensiveness, which was linked to the high quality of resources provided. Some teachers noted that they had used the training materials in school. For example, during Mental Health Awareness week, one school had shared a video to teachers and students:

“Yes, I have used the videos for example with my form teachers to try and get across to the other children how important mental health is, just to show them particularly the one with the boy standing up on the chair and the black dog. We use them at registration time. (Teacher, One-Day MHFA for Schools and Colleges trained Teachers, School 3P, Wales, Phase 2)”

The second dimension of acceptability was self-efficacy to deliver the intervention. A number of attendees maintained that they had developed new knowledge, awareness, and skills, commenting that the training was relevant and appropriate:

“I thought it was very good and it was very practical. It was very kind of top line. We had Kids Company come in, and it was on a par with that, in that we felt like we learnt something. (Peer Supporter, Standard MHFA Training, School 1D England, Phase 2)”

Some participants also felt that the training both reinforced and consolidated existing practices within the school, giving them more confidence to implement them:

“Yes, I know that somebody who did receive the training has been using that training in conversations with other members of staff, but I think that she was doing that anyway... having that training has probably done what it did for us, which is just give that extra bit of confidence…(Teacher, MHFA for Schools and Colleges trained Teachers, School 3P Wales, Phase 1)”

There was scope to improve confidence to deliver support, with some participants recommending more of a focus on skills and knowledge in relation to common mental health issues that might be directly linked to workplace stress in schools:

“A lot of the training was about things like recognising schizophrenia and extreme depression, what I felt was missed was just the everyday mental health issues, stress. We did touch on stress, but that is what nine times out of ten, people are going to be suffering from here, and anxiety. (Peer Supporter, Standard MHFA Training, School 1D England, Phase 2)”

The third dimension of acceptability, and more in relation to the peer support service, was a belief that the intervention could be effective. It was primarily headteachers who commented that the intervention could provide a useful system to identify and address issues related to staff wellbeing:

“I think the peer training that took place at the start of this term, to have staff in school who are accessible for other staff within school and not members of the Leadership Team I think gives us a far better conduit to deal with issues than perhaps otherwise would have been the case. (Headteacher, School 4N, Wales)”

Teaching staff also observed that the upskilling of colleagues gave them more confidence in the pastoral support that they were offering:

“I think having those people that … Because the people who were on the training were identified by us as well, we put their names down, they were the people that already we felt that people were going and speaking to them anyway, and just knowing now that they have that little bit of training behind it as well, that doesn’t hurt. (Teacher, One-Day MHFA for Schools and Colleges trained Teachers, School 3P, Wales, Phase 1)”

Drawing together data on the dimensions of acceptability, largely decontextualized and before the intervention coupled with the school system, there was some evidence of positive perceptions. Equally, there were opportunities for improvement. This acceptability became more ambiguous, nuanced and even problematic however as components were understood within the context of the complex social system of schools (Table 1).

**Table 1:** Acceptability of WISE intervention: overview of main themes and sub-themes

**Main theme**	**Sub-theme**	**Description of sub-theme**
Acceptability of WISE intervention components	Affective attitudes	Positive and excited attitude towards intervention components
	Coherency and comprehensibility	High quality, comprehensive training resources (e.g. videos and materials)
	Self-efficacy	Intervention develops new knowledge, awareness, and skills that are contextually relevant. Reinforced and consolidated existing practice. Could focus more on common mental health disorders, particularly in relation to workplace stress
	Perceived effectiveness	Could provide a useful system to identify and address staff mental health and wellbeing
Acceptability of WISE intervention components when interacting with school system: structural drivers of mental health and wellbeing not disrupted	Complex student and staff needs	Increased staff responsibility for complex student needs due to declining availability of external support services. Peer support inadequate in supporting complex staff needs
	Culture of mistrust and stigma around disclosing mental health problems	Culture of mistrust meaning confidentiality cannot be maintained. Fear of negative judgement and punitive treatment when disclosing mental health concerns. Exacerbated by stigma around mental health and the isolated nature of teaching
	Workload and accountability	Problematic and unmanageable workload. Compounded by stringent accountability and inspection processes

### Acceptability of WISE Intervention in a Complex Social System

As the WISE intervention was experienced and considered in relation to the wider school structures, there were more critical perceptions amongst participants, particularly teaching staff.

Reflecting on the system’s starting point for engaging in the WISE study, schools’ rationale for participation, which was mainly expressed by headteachers, seemed linked to them reaching a ‘tipping point’ and becoming orientated to change. There were three key markers of this ‘tipping point’: recognition that teacher mental health had been neglected previously; a political climate of ‘austerity’ increasing pressure on teachers; and a growing emphasis in public health policy on the need to consider teachers as part of a whole-school approach to mental health.

First, schools’ wellbeing agenda was considered to historically be restricted to students, with staff expressing frustration at the lack of resources available to them:

“I’m diagnosed with depression, and I actually went to the school nurse and the school counsellor and said, is there any opportunity for me to have some counselling? They were like, oh you can phone this number or such and such, go and see your GP. But it was, no, you can’t come and talk to me, only the children can. I thought, that’s a shame*.* (Peer Supporter, Standard MHFA Training, School 2L, England, Phase 1)”

More recently, the agenda was expanding to include teacher mental health. Contributory factors included increased concern about teachers’ ability to fulfil their role when experiencing poor wellbeing, coupled with the growing risk of burnout and withdrawal from the profession. Headteachers outlined the value of investing in teachers’ health:

“You can’t have jobs where there’s an expectation at some point you burn out, that’s not sustainable. … In terms of retention, recruitment, every profession has got to look after people who, you’ve got to enjoy your job. (Headteacher, School 1I, England)

We are an outcomes driven organisation but it is about ensuring that the workforce feel valued and if the workforce feels valued and supported, the likelihood is that they are more likely then to fulfil the goals of the organisation. (Headteacher, School 4N, Wales)”

Second, participants suggested that teachers need good mental health literacy to effectively support student wellbeing and academic performance. There was acknowledgement of the current climate of ‘austerity’, where sustained and increasing resource cuts were reducing the availability of external support. Headteachers observed that teachers were now shouldering this burden, and wanted to implement interventions that could help them in this task:

“…how schools move to up-skill staff now is absolutely vital…in terms of the wellbeing of students.... we’re not going to be able to employ, because we haven’t got the budget, a raft of educational psychologists. So, it’s whether or not there are certain interventions or certain training schemes. (Headteacher School 4U, Wales)”

Third, within the wider educational and health system, there was indication that whole school approaches to mental health, that included teacher wellbeing, were being actively encouraged. Representatives from public health agencies who funded the intervention stated that it aligned with their strategic vision and direction of travel:

“Yes, it [WISE intervention] fits well. We have also relaunched our healthy schools programme and the mental health badge, which enables schools to just focus on particularly mental health, so that they can really dedicate their time to that. (Funder One, England)”

In the initial period of WISE adoption then, and at a time where schools had seemingly reached a tipping point, stakeholders felt positively orientated towards the intervention and its potential in the system. Reflecting on the introduction of the intervention, some felt it had initially been a symbolic response to the need for transformation:

“… the staff body certainly felt like they had, that the school was paying some attention. (Peer Supporter, Standard MHFA Training, School 1D, England, Phase 2)”

However, as the WISE intervention began to interact with the complex school system, acceptability became more equivocal. This is partly supported by the implementation data, published elsewhere (Fisher et al., 2020), where there was low uptake of the peer support service. There were also emergent concerns, particularly amongst teachers and peer supporters, that the intervention was minimally disruptive of the structural determinants of poor teacher wellbeing. This was despite these structural determinants not being within the remit of the programme theory. In consequence, it was deemed that the intervention impact would ultimately be negligible. One peer supporter in particular reflected how the intervention had not sufficiently disrupted entrenched system practices:

“Yes, it’s much better to offer support, but the context … That’s the challenge, it’s trying to think oh yes, I’m trying to get the best out of people’s performance and everything else. … You understand, but whether your line manager will understand … So it needs empathy throughout the system really, to give you a bit of space. (Peer supporter, Standard MHFA Training, School 4N, Wales, Phase 2)”

From participant accounts, there were three central structural drivers of poor teacher mental health that the intervention did not address.

#### Complex Student and Staff Needs

From the outset, school staff reflected that students were displaying increasingly complicated and challenging behaviors within the school context, which were attributed to disrupted family structures, peer groups, and social media. Coupled with the declining availability of external support services, staff recognised that they had a growing responsibility in meeting these complex needs, which could adversely affect their own mental health. Some individuals expressed concern from the study start that the intervention could not be a panacea for students’ problems, as it did not provide adequate training or skills and the role of the teacher was not intended to provide mental health support:

“I’m glad it’s being addressed because I think with services being cut and less options to have to refer students, I can see why they’re making, like putting it in schools because they’re kind of replacing services... But I think it needs to be really thought of carefully, in terms of in peer support, how is that going to work and does it work? And if it’s going to be a replacement service for students, then it needs to be a lot tighter, a lot more training. (Teacher, One-Day MHFA for Schools and Colleges trained Teachers, School 1D, England, Phase 1)”

Participants further considered these issues in relation to meeting the needs of colleagues. Even though peer supporters were not expected to offer counselling, there was concern that they would be forced to address the complex emotional needs of staff without any professional skill:

“it has a horrible element of box ticking about it, in that you’re asking people who aren’t qualified, it was a great day we did, but they’re not qualified other than being on a couple of days training, professionally qualified to talk about mental health. (Teacher, One-Day MHFA for Schools and Colleges trained Teachers, School 1D, England, Phase 1)”

Perhaps more significantly, a number of participating teachers indicated that focusing on teachers’ competency in supporting staff and student mental health was merely detracting from the real systemic issue, which was the dearth of external educational and mental health services.

“And as much as we can do what we can do, we need more time, we need more counsellors… There are things that the school could do but there’s nobody out there, and it needs better access to external services. (Peer Supporter, Standard MHFA Training, School 3P, Wales, Phase 1)”

#### Culture of Mistrust and Stigma Around Disclosing Mental Health Problems

The WISE intervention necessitates that the peer support service is confidential, and schools were actively encouraged to adopt formalised confidentiality agreements so that individuals who use the service are clear on how their information would be handled. However, from the outset, a number of participating teachers expressed concern that confidentiality could not be maintained. This was in part because some schools did not draw up a protocol. It was also a response to a wider culture of mistrust that participants, notably teachers, felt characterised their school:

“it’s such a high risk to take if it’s not confidential or if someone just says something without thinking…. If you want to talk about mental health problems, you should really go to a professional I think, particularly in the environment we work in, where everyone knows pretty much everything. (Teacher, One-Day MHFA for Schools and Colleges trained Teachers, School 1D, England, Phase 1)”

Such sentiments only seemed to strengthen as the study progressed:

“I have absolutely no confidence whatsoever in confidentiality within schools. I know lots of things I’m not supposed to know, I’m sure that many other people do too... Several of the people on there [list of peer supporters] I think would have told other people, so I didn’t talk to them... I did email one person and we were going to meet but then I was actually worried about people seeing me with her and then being, well you would never, what reason do you have to speak to her, and putting two and two together, so I just didn’t speak to her. (Teacher, One-Day MHFA for Schools and Colleges trained Teachers, School 1D, England, Phase 2)”

Within these accounts, concerns were expressed about the consequences of disclosing mental health problems, or having others find out about such problems as a result of broken confidentiality. A number of participants spoke about the vulnerability experienced when previously sharing problems with colleagues:

“I think it’s frowned upon for me, if I take my mental health issues to my employer and they kind of fob it off and they’re not bothered, it just left me, that’s what happened, and then it left me in quite an insecure place. I felt like I was very much on my own with it and I felt quite paranoid... (Teacher, One-Day MHFA for Schools and Colleges trained Teachers, School 1D, England, Phase 2)”

In initial focus groups, some participants stated that they would be negatively judged if they disclosed that they were struggling to manage their workload, and this remained a common theme throughout:

“I do feel in this job it’s a judge culture, and I don’t like to admit when I’m struggling and when I’m finding things really difficult, or difficult to complete, or whether I’m able to keep up on marking. So whilst it’s there [WISE Intervention], … I always have that in the back of my mind. (Teacher, MHFA for Schools and Colleges trained Teachers, School 4N, Wales, Phase 1)”

Furthermore, participants commented that mental health was stigmatised within the wider culture, and the WISE intervention was trying to operate within a set of entrenched norms where individuals do not discuss personal issues in a professional environment. One peer supporter recounted an incident where they had offered pastoral support to a colleague, but this had been negatively appraised by their line manager as they felt it was inappropriate to openly discuss such issues:

“And I was making suggestions [to support colleague], and the line manager had a few pops at me, in terms of, well it’s quite clear you’re supporting that member of staff. (Peer Supporter, Standard MHFA Training, School 2L, England, Phase 2)”

Another participant recalled a colleague being disciplined because they had discussed their mental health with students:

“he was suspended for it unofficially for several days because he had discussed his mental health issues. Well hang on a second, we are supposed to be open about this now, we are doing all of this and yet it is still supposed to be swept under the carpet. (Teacher, One-Day MHFA for Schools and Colleges trained Teachers, School 3P, Wales, Phase 2)”

There were a number of factors that contributed to this culture of mistrust, with one of the most prominent being the isolated nature of teaching. Participants maintained that it was difficult to build a community with their colleagues. This is partly a consequence of being alone in the classroom for prolonged periods of time, school layouts (e.g. large, sprawling campuses) that inhibited communal activity, the declining use of traditional communal spaces (e.g. staff rooms), and the irregularity of social activities:

“That’s actually a really important point because when you’re teaching you are on your own. And if you’re having like a bad day or an emotional day or it’s all going wrong, you’re in a room and you cannot leave that room because you can’t leave those young people alone. And so you’re completely exposed because it’s a job where you’re standing up in front of people and vulnerable. (Peer Supporter, Standard MHFA Training, School 2L, England, Phase 2)”

#### Workload and Accountability

The high workload of teachers was considered problematic, with many finding it to be unmanageable. A number of school staff commented on the stress and overwhelm that they encountered on a daily basis:

“I sat at my desk on Monday or Tuesday morning and I just had so much, literally, so many things to do, I almost got to the point where I just couldn’t function which way to go, because there was just so many things. And it just gets added to and added to and added to. (Teacher, MHFA for Schools and Colleges trained Teachers, School 2L, England, Phase 1)”

This issue was seen to be compounded by stringent accountability and inspection processes, which not only served to increase workload and pressure, but reduced the feeling of autonomy within teachers’ role. One participant felt that the constant inspection meant they were not trusted to carry out their work to a professional standard when unsupervised, suggesting tension between senior leadership and staff:

“One part of teaching is that constant… I think we’re constantly being assessed, so through book scrutiny, that element of not trusted and therefore leadership needs or must check that we’re doing what we’re meant to be doing, and I don’t like that side of the job. (Teacher, MHFA for Schools and Colleges trained Teachers, School 4N, Wales, Phase 1)”

The wider context of workload and monitoring structures left some school staff frustrated at the intervention. This was partly due to it actually adding to already burdensome workloads. One trainer reflected that even during the training sessions, attendees expressed concern about their capacity to deliver the intervention:

“when we’re talking, like I went into, one part of it was about Five Ways to Wellbeing, so we talk about raising mental health, and one of the teachers just literally threw at me, so when do you think we’re going to have the time for that?” (Trainer 2)”

It further left some peer supporters with a sense of inadequacy in their new role within the intervention. In some instances, there was even a sense that the peer supporter role could be unintentionally harmful to wellbeing, as it might instigate anger and hopelessness about the seemingly intractable structural determinants of wellbeing that were not being addressed:

“But I feel like people would come to me with problems and they would just make me more angry about the situation that I’m in and the fact that I can’t change it. (Teacher, MHFA for Schools and Colleges trained Teachers, School 1D, England, Phase 2)”

In summary, the acceptability of the WISE intervention was adversely impacted by the continued presence of the structural drivers of poor mental teacher mental health. While the WISE intervention did not claim to target these system level causes, participants felt that its limited potential to modify such structural conditions made it an inadequate approach.

## Discussion

The present paper reports the acceptability of the WISE intervention to stakeholders. This was to support explanation of the limited effectiveness reported by the outcome evaluation (Kidger et al., 2021). Drawing on a complex systems perspective (Hawe et al., 2009; Moore et al., 2018), the study explored the contextual contingency of different participants’ experiences, and how largely acceptable components lost their appeal when seen as being minimally disruptive of wider system structures. Indeed, while the school system was arguably at a ‘tipping point’ at study commencement (Gladwell, 2006), and orientated to changing practices in order to address the problem of teacher mental health and wellbeing, hostility and resentment emerged as participants’ recognised that wider systematic drivers were not being addressed. This was despite it being clear from the outset that the intervention’s theory of change did not directly target these factors. 

Reported elsewhere, these system drivers were as follows: complex student and staff needs; high workload and stringent accountability; and a culture of mistrust which was exacerbated by the stigma relating to mental health (Barmby, 2006; Skaalvik & Skaalvik, 2017). This finding may part explain the lack of intervention impact, potentially via the low uptake of the peer support service (Fisher et al., 2020). Study participants stated they did not need it, and this may be because it does not tackle the perceived causes of poor mental health.

The study provides a number of important lessons for intervention evaluation, and how acceptability is understood and assessed. Central to these lessons is the fact that the WISE intervention was largely acceptable at the piloting stage (Kidger et al., 2016b), and it was only during the effectiveness RCT that the presented issues emerged. This suggests that while frameworks that emphasise the acceptability of components have utility (Sekhon et al., 2017; Weiner et al., 2017), it is important to ground understanding of participants' perceptions in the context in which they are experienced (Moore et al., 2015). As such, it is imperative that acceptability be seen as a constantly emergent and dynamic construct that evolves as individuals interact with the intervention in a changing system. It should be explored through a mixed method approach that can capture complexity (Moore et al., 2018).

Relatedly, there is also a need to explore acceptability at all stages of evaluation. There can be a propensity to consider it in detail only at the feasibility and piloting phases of intervention evaluation, and if it meets the progression criterion, it is assumed in the full effectiveness evaluation. However, the period of feasibility testing is often short, and the complex experiences and perceptions of the intervention may unfold beyond this timeframe. While this evolution may involve a decline in acceptability, it is important to recognise that an intervention may also meet resistance within the system initially, before being fully assimilated as understanding of its merit evolve.

Participants’ perceptions and experiences of the WISE intervention provide useful guidance for the future development of interventions in relation to teacher mental health and wellbeing. In particular, the data resonate with previous critiques of public health interventions that warn against negligible or negligent public health approaches that merely tinker at the edges rather than addressing the central determinants of a problem (Hawe, 2015).

System-based approaches need to be progressed. This may mean extending whole school-approaches focused on student health outcomes to include teachers’ health more explicitly, even though the evidence-base for such interventions is mixed (Goldberg et al., 2019; Langford et al., 2017). Interventions may also focus on combining individual or inter-personal activities (Bartlett et al., 2019; von der Embse et al., 2019), with a degree of contextual restructuring to ensure they can gain traction. In particular, there needs to be a focus on implementation strategies and ensuring sustained and meaningful engagement from senior leaders to ensure that intervention is not tokenistic. 

However, while we suggest these avenues for intervention development, further research is still needed to systematically map teachers’ own understanding of the determinants of their mental health and their views on the solution to their support needs. This is to ensure that the theoretical basis of these interventions is aligned with the drivers of the problem that dominate in local contexts. Community-based participatory approaches have much to offer in supporting the engagement of diverse stakeholders in this process (Greenhalgh et al., 2019; Jull et al., 2017).

There are important implications for policy and practice. Within the UK context, there is a paucity of approaches to improve teacher mental health, although there have been recent efforts to upskill school staff in relation to promoting student wellbeing. For example, the UK Government announced the roll-out of Mental Health First Aid training for a minimum of one teacher per secondary school across England (Prime Minister’s Office, 2017). However, it is debatable whether this is sufficient or acceptable, or may even lead to harms, especially in light of the findings from this study.

There have been some attempts at system-level changes. Schools in Wales are currently engaging in a process of curriculum reform, which seeks to provide a holistic approach to education that privileges health and wellbeing (Donaldson, 2015; Welsh Government, 2021). In England, there is the rollout of trailblazer sites, focused on creating stronger support for mental health for young people within schools, and links between schools and Child and Adolescent Mental Health Services (Ellins et al., 2019), which might alleviate some of the stressors that school staff experience. The Department for Education continues to provide guidance to leaders in order to support school staff and redress structural issues, particularly in relation to reducing workload (Department for Education, 2019). Yet despite the potential of such reforms, there remains a lack of provision to support teachers’ mental health alongside that of students. This is particularly important as teachers are often required to implement student-level reforms, and transform existing ways of working. To this end, policy needs to foreground provision to support and monitor teachers’ wellbeing within services, curricula and inspectorate frameworks.

## Limitations

First, while data were generated across two phases, allowing some exploration of the dynamic nature of acceptability, they were not collected from all participants at each timepoint. This was due to a decision to focus on key areas of uncertainty at each time point (e.g. the study was keen to explore headteacher buy-in at the initial stages of adoption and implementation). Hence, the results present more of a study level overview of changes to acceptability, rather than individual-level changes over the course of the evaluation. Second, most process data came from the four case-study intervention schools. Third, data from peer supporters and teachers were primarily conducted through focus groups. While this helped to elicit important group and system-level dynamics, they had the potential to constrain participants’ willingness to share, especially given the documented culture of mistrust and stigma around mental health. Fourth, data were largely collected from school-level stakeholders and further insight could have been provided by the wider educational system.

## Conclusion

Teacher mental health and wellbeing is an important issue, but current interventions are limited to individually focused approaches, which have a relatively weak evidence base. The WISE intervention aimed to provide a more comprehensive approach through peer support. The intervention did not report effectiveness. This paper demonstrates how examination of acceptability is imperative to understanding outcomes. In the case of the WISE intervention, acceptability declined as the intervention interacted with complex system dynamics over time. Moving forward, there is a need for a more nuanced and complex system-based approach to acceptability to maximise the likelihood of sustainable intervention implementation and effectiveness.
